# Trackplot: a fast and lightweight R script for epigenomic enrichment plots

**DOI:** 10.1093/bioadv/vbae031

**Published:** 2024-02-28

**Authors:** Anand Mayakonda, Frank Westermann

**Affiliations:** Hopp Children’s Cancer Center (KiTZ), 69120 Heidelberg, Germany; Division of Neuroblastoma Genomics, German Cancer Research Center (DKFZ), 69120 Heidelberg, Germany; Hopp Children’s Cancer Center (KiTZ), 69120 Heidelberg, Germany; Division of Neuroblastoma Genomics, German Cancer Research Center (DKFZ), 69120 Heidelberg, Germany

## Abstract

**Motivation:**

BigWig files serve as essential inputs in epigenomic data visualization. However, current R packages for visualizing these files are limited, slow, and burdened by numerous dependencies.

**Results:**

We introduce *trackplot*, a minimal R script designed for the rapid generation of integrative genomics viewer (IGV) style track plots, profile plots, and heatmaps from bigWig files. This script offers speed, owing to its reliance on *bwtool*, resulting in performance gains of several magnitudes compared to equivalent packages. The script is lightweight, requiring only the *data.table* and *bwtool* packages as primary dependencies. Notably, the plots are generated in base R graphics, eliminating the need for additional packages. *trackplot* queries the University of California Santa Cruz (UCSC) genome browser for gene models thereby enhancing the reproducibility of analyses. The script extends its support to general transfer format (GTF) further enhancing its versatility. This tool addresses the gaps in existing bigWig visualization approaches by offering speed, simplicity, and minimal dependencies, thereby presenting a valuable asset to researchers in the fields of epigenomics.

**Availability and implementation:**

*trackplot* is implemented in R is made available under MIT license at https://github.com/PoisonAlien/trackplot.

## 1 Introduction

Efficient data visualization is a critical step in the analysis of epigenomics and transcriptomics, enabling the interpretation of complex biological insights. These analyses often demand the representation of quantitative variations across genomic locations and experimental conditions. Notably, sequencing methodologies like chromatin immunoprecipitation with sequencing (ChIP-seq), assays for transposase-accessible chromatin with sequencing (ATAC-seq), and whole genome bisulfite sequencing (WGBS) produce voluminous data, underscoring the significance of effective visualization tools. BigWig files have emerged as essential components of data representation (Kent *et al.* 2010). These binary, compressed, and indexed files offer rapid data accessibility, making them the preferred choice for storing extensive datasets. As such, they also constitute the primary data source for visualization.

However, the current landscape of data visualization tools in R such as *Gviz* and *karyoploteR* comes with limitations (Hahne and Ivanek 2016, Gel and Serra 2017). These tools often suffer from cumbersome dependencies and speed when handling large datasets.

To address these limitations, we introduce *trackplot—*an R- script that takes a lightweight and efficient approach, leveraging *bwtool* (Pohl and Beato 2014) to significantly enhance data extraction from bigWig files, and base R graphics for complex visualizations. Notably, *trackplot* significantly minimizes dependencies, simplifying installation and maintenance. This provides an elegant solution for streamlined epigenomic data analyses.

## 2 Results

### 2.1 Lightweight and minimal dependency

**Figure 1.**
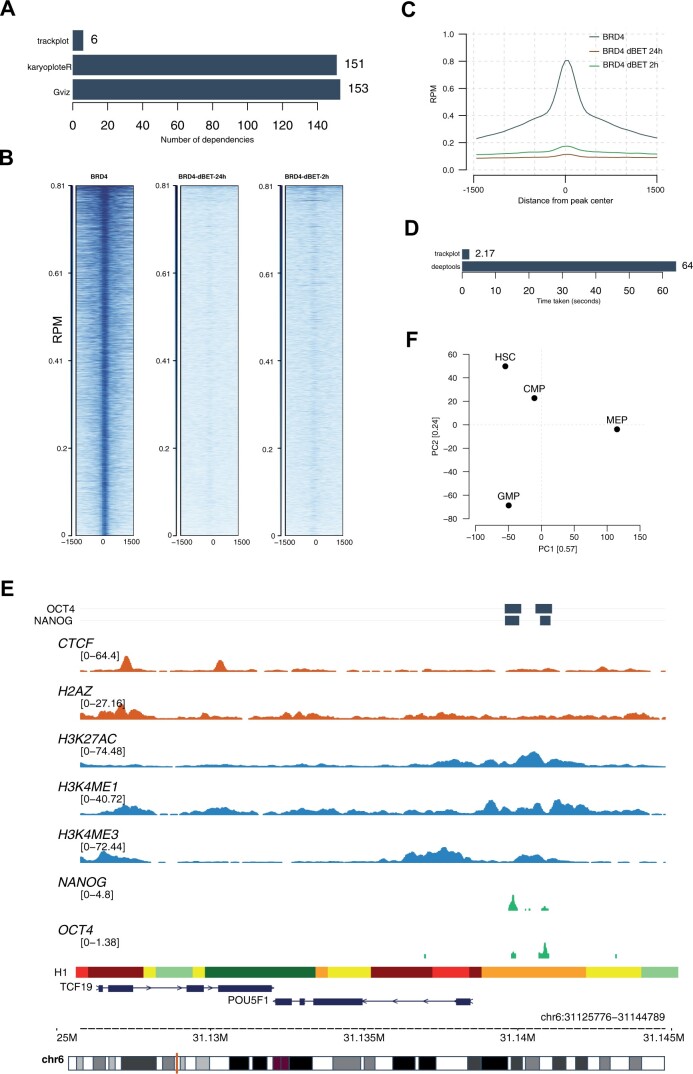
(A) Number of dependency packages required to install *trackplot, karyoploteR*, and *Gviz* R packages (lower is better). (B) Heatmap and (C) Profile plot for BRD4 binding sites in U87 cell lines treated with DMSO and BRD4 degrader at 2 and 24 hours respectively (data from GSE99171 (Xu *et al.* 2018)). (D) Time taken by *trackplot* and *deeptools* to generate (B) and (C). (E) ENCODE ChIP-seq data for human embryonic stem cell line H1 visualized as an IGV style track. The top panel shows OCT4 and NANOG binding sites. The bottom plots show chromHMM models, gene models, and cytoband extracted from the UCSC genome browser. (F) Principal component analysis of hematopoietic stem and myeloid progenitor cells based on the H3K27Ac signal across the promoters of the protein-coding genes (1200 bp upstream and 800 bp downstream of transcription start sites). The plot shows the standard differentiation trajectory of HSCs on principal component 2 (top to bottom). Data was obtained from GEO accession number GSE231426 (Subramanian *et al.* 2023). HSC: Haemopoietic stem cells; CMP: Common myeloid progenitor; MEP: Megakaryocte-Erythroid Progenitor cells; GMP: Granulocyte monocyte progenitor cells.

*trackplot* leverages the *data.table* R package for rapid data import and manipulation, utilizing *bwtool* to process bigWig files of queried regions. Dynamic SQL queries enable the extraction of gene models from the UCSC genome browser (Nassar *et al.* 2023), and complex visualizations are executed using base R graphics, circumventing heavy dependencies like *ggplot2* and *tidyverse* packages—that often suffer from version compatibility issues, external system dependencies, longer run-times, and frequent installation errors. These design choices result in swift processing and a lightweight package structure suitable for UNIX-based systems—commonly used in bioinformatic analysis. Comparative analyses of package dependencies, in contrast to similar R packages such as *Gviz*, and *karyoploteR*, underscore the advantages of *trackplot* (Fig. 1A) (Gu and Hübschmann 2022). Its efficient, dependency-light approach facilitates accelerated analysis and visualization workflows.

### 2.2 Profile plots and heatmaps for epigenomic datasets

*tracklplot* allows generating profile plots and heatmaps, two fundamental visualizations in epigenomic datasets such as ChIP-seq and ATAC-seq (Fig. 1B and C). Leveraging the *bwtool*, the script extracts the underlying signal for user-defined genomic regions from BED files or can automatically retrieve transcript models from the UCSC genome browser. Comparing *trackplot* with *deeptools* (Ramírez *et al.* 2014) – a commonly used software for processing bigwig files, showed that *trackplot* is up to 20 times faster for generating profile plots and heatmaps from the same dataset (Fig. 1D). Of note, extracting signals from bigwig files across thousands of genomic regions is often computationally expensive and time consuming. Trackplot significantly reduces the run times while offering additional functionalities. This speed is primarily owed to the efficient processing of bigwig files by *bwtool* which is further enhanced by rapid aggregation and plotting of the resulting output in R. Please refer to the Supplementary Material for the details on the datasets, reproducible code, and bench-marking experiments.

### 2.3 Visualizing IGV style tracks

Key visualization in *trackplot* is a user-friendly mechanism for generating IGV (Integrative Genomics Viewer) style signal tracks (Fig. 1E). This functionality allows for the extraction and visualization of tracks from either specific genomic regions of interest or individual target genes. The script extracts gene models for the queried regions from the UCSC genome browser database by employing dynamic SQL queries. Additionally, the tool extends its capabilities to incorporate chromHMM data visualization. chromHMM models can be from user-provided datasets or loaded from 20 cell lines accessible via the UCSC genome browser. The option to incorporate custom gene models in GTF format adds a layer of flexibility to the visualization process. Moreover, the plotting function enables customization such as highlighting target loci of interest, summarizing replicates per condition, and auto-scaling the tracks. Notably, in the absence of bigWig files the script accommodates the use of narrowPeak or broadPeak files to depict peak intensities.

### 2.4 Comprehensive insights through summary plots and PCA

In addition to the plotting functions, *trackplot* can be employed to extract peak intensities, thereby enabling a quantitative overview of genomic loci across conditions. In particular, for uniformly processed and normalized bigWig files, these values can be utilized for dimensional reduction such as principal component analysis (PCA; Fig. 1F).

## 3 Conclusion

Although *trackplot* does not implement any novel algorithms or data structures, it excels in bringing together existing solutions to provide an easy-to-use toolset—implemented in R programming language. Overall, *trackplot* offers an elegant data visualization solution for epigenomic datasets. Its versatile functionalities can be used for various analysis needs, ranging from IGV-style genomic tracks to profile plots, heatmaps, summary plots, and PCA. Furthermore, by mitigating the heavy R package dependencies, *trackplot* demonstrates its potential in data visualization while offering speed and ease of use.

## Supplementary Material

vbae031_Supplementary_Data

## Data Availability

All the data sources used are properly cited with the accession numbers wherever required.
