# Supplementary material for: Trackplot: a fast and lightweight R script for epigenomic enrichment plots
Source: Bioinform Adv. 2024 Feb 28;4(1):vbae031. doi: 10.1093/bioadv/vbae031 (PMC10932608; doi:10.1093/bioadv/vbae031)
Supplement: vbae031_Supplementary_Data [file vbae031_supplementary_data.zip › Supplementary_data/trackplot.html]

Supplementary data


# Supplementary data

This file contains code used for generating results for the
manuscript **Trackplot: A fast and lightweight R script for
epigenomic enrichment plots**

## Dependency

`trackplot` requires bwtool to be
installed and accessible from the command line.

1. For macOS: Please download the pre-built binary from here

Make it executable with `chmod +x bwtool`. macOS
gatekeeper might complain that it can’t run the binary downloaded from
the internet. If so, allow it in the
security settings.

2. For centOS or debian: Follow these compilation
   instructions.

Finally move the `bwtool` binary to a PATH (e.g;
/usr/local/bin) or to a directory under the PATH. Alternatively, you
could also add the path where bwtool is located to R session with the
below command.

```
#Example
Sys.setenv(PATH = paste("/Users/anand/Documents/bwtool_dir/", Sys.getenv("PATH"), sep=":"))
```

## Load the code

```
source("trackplot.R")

# OR If you prefer to install it as a package
# remotes::install_github(repo = "poisonalien/trackplot")
```

See trackplot
github repo for more installation options.

## IGV style track plots

### Download ENCODE H1 data

Download bigWig files for H1 - Human Embryonic Stem Cell derived cell
lines from ENCODE.

```
dir.create(path = "data/H1/", showWarnings = FALSE, recursive = TRUE)

options(timeout = max(300, getOption("timeout")))

#TFs
download.file("http://hgdownload.soe.ucsc.edu/goldenPath/hg19/encodeDCC/wgEncodeHaibTfbs/wgEncodeHaibTfbsH1hescPou5f1sc9081V0416102RawRep1.bigWig", destfile = "data/H1/OCT4.bw", quiet = TRUE)
download.file("http://hgdownload.soe.ucsc.edu/goldenPath/hg19/encodeDCC/wgEncodeHaibTfbs/wgEncodeHaibTfbsH1hescNanogsc33759V0416102RawRep1.bigWig", destfile = "data/H1/NANOG.bw", quiet = TRUE)

#Histones
download.file("http://hgdownload.soe.ucsc.edu/goldenPath/hg19/encodeDCC/wgEncodeBroadHistone/wgEncodeBroadHistoneH1hescH2azStdSig.bigWig", destfile = "data/H1/H2AZ.bw", quiet = TRUE)
download.file("http://hgdownload.soe.ucsc.edu/goldenPath/hg19/encodeDCC/wgEncodeBroadHistone/wgEncodeBroadHistoneH1hescCtcfStdSig.bigWig", destfile = "data/H1/CTCF.bw", quiet = TRUE)
download.file("http://hgdownload.soe.ucsc.edu/goldenPath/hg19/encodeDCC/wgEncodeBroadHistone/wgEncodeBroadHistoneH1hescH3k4me3StdSig.bigWig", destfile = "data/H1/H3K4ME3.bw", quiet = TRUE)
download.file("http://hgdownload.soe.ucsc.edu/goldenPath/hg19/encodeDCC/wgEncodeBroadHistone/wgEncodeBroadHistoneH1hescH3k27acStdSig.bigWig", destfile = "data/H1/H3K27AC.bw", quiet = TRUE)
download.file("http://hgdownload.soe.ucsc.edu/goldenPath/hg19/encodeDCC/wgEncodeBroadHistone/wgEncodeBroadHistoneH1hescH3k4me1StdSig.bigWig", destfile = "data/H1/H3K4ME1.bw", quiet = TRUE)

#Peak files for TFs
download.file("http://hgdownload.soe.ucsc.edu/goldenPath/hg19/encodeDCC/wgEncodeHaibTfbs/wgEncodeHaibTfbsH1hescPou5f1sc9081V0416102PkRep1.broadPeak.gz", destfile = "data/H1/OCT4.bed.gz", quiet = TRUE)
download.file("http://hgdownload.soe.ucsc.edu/goldenPath/hg19/encodeDCC/wgEncodeHaibTfbs/wgEncodeHaibTfbsH1hescNanogsc33759V0416102PkRep1.broadPeak.gz", destfile = "data/H1/NANOG.bed.gz", quiet = TRUE)
```

### Prepare coldata

```
h1_bigWigs = list.files(path = "data/H1/", pattern = "\\.bw$", full.names = TRUE)
h1_bigWigs = read_coldata(bws = h1_bigWigs, build = "hg19")
```

```
## Checking for files..
```

```
## Input type: bw
```

```
## Ref genome: hg19
```

```
## OK!
```

```
h1_bigWigs
```

```
##               bw_files bw_sample_names
##                 <char>          <char>
## 1:    data/H1//CTCF.bw            CTCF
## 2:    data/H1//H2AZ.bw            H2AZ
## 3: data/H1//H3K27AC.bw         H3K27AC
## 4: data/H1//H3K4ME1.bw         H3K4ME1
## 5: data/H1//H3K4ME3.bw         H3K4ME3
## 6:   data/H1//NANOG.bw           NANOG
## 7:    data/H1//OCT4.bw            OCT4
```

### Exctract signal

```
#Region to plot
oct4_loci = "chr6:31125776-31144789"

#Extract bigWig signal for a loci of interest
t = track_extract(colData = h1_bigWigs, loci = oct4_loci)
```

```
## Parsing loci..
```

```
##     Queried region: chr6:31125776-31144789 [19013 bps]
```

```
## Querying UCSC genome browser for gene model and cytoband..
```

```
## Extracting gene models from UCSC:
##     chromosome: chr6
##     build: hg19
##     query: mysql --user genome --host genome-mysql.soe.ucsc.edu -NAD hg19 -e 'select chrom, txStart, txEnd, strand, name, name2, exonStarts, exonEnds from refGene WHERE chrom ="chr6"'
```

```
## Extracting cytobands from UCSC:
##     chromosome: chr6
##     build: hg19
##     query: mysql --user genome --host genome-mysql.soe.ucsc.edu -NAD hg19 -e 'select chrom, chromStart, chromEnd, name, gieStain from cytoBand WHERE chrom ="chr6"'
```

```
## Generating windows [10 bp window size]
```

```
## Extracting signals
```

```
##     Processing CTCF ..
```

```
##     Processing H2AZ ..
```

```
##     Processing H3K27AC ..
```

```
##     Processing H3K4ME1 ..
```

```
##     Processing H3K4ME3 ..
```

```
##     Processing NANOG ..
```

```
##     Processing OCT4 ..
```

```
## OK!
```

### Plot

```
track_cols = c("#d35400","#d35400","#2980b9","#2980b9","#2980b9", "#27ae60","#27ae60") #Some pretty colors for tracks
oct4_nanog_peaks = c("data/H1/NANOG.bed.gz","data/H1/OCT4.bed.gz") #Peak files 

track_plot(
  summary_list = t,
  col = track_cols,
  show_ideogram = TRUE,
  genename = c("POU5F1", "TCF19"),
  peaks = oct4_nanog_peaks,
  peaks_track_names = c("NANOG", "OCT4"),
  groupAutoScale = FALSE, ucscChromHMM = "wgEncodeBroadHmmH1hescHMM", y_min = 0
)
```

```
## Extracting chromHMM from UCSC:
##     chromosome: chr6
##     build: hg19
##     query: mysql --user genome --host genome-mysql.soe.ucsc.edu -NAD hg19 -e 'select chrom, chromStart, chromEnd, name from wgEncodeBroadHmmH1hescHMM WHERE chrom ="chr6"'
```

```
## Collapsing transcripts..
```

## Dependency heaviness

Number of R dependency packages required by karyoploteR
and Gviz
- two packages which facilitate IGV like track visualization.

Use pkgndep
to measure the dependency heaviness.

```
library(pkgndep)
```

```
## ========================================
## pkgndep version 1.99.3
## CRAN page: https://CRAN.R-project.org/package=pkgndep
## Github page: https://github.com/jokergoo/pkgndep
## Documentation: https://jokergoo.github.io/pkgndep/
## 
## If you use it in published research, please cite any of them:
## Gu, Z. Pkgndep: a tool for analyzing dependency heaviness 
##   of R packages. Bioinformatics 2022.
## Gu, Z. On the dependency heaviness of CRAN/Bioconductor 
##   ecosystem. Journal of Systems and Software 2023.
## 
## This message can be suppressed by:
##   suppressPackageStartupMessages(library(pkgndep))
## ========================================
```

```
#get Dependency heaviness with pkgndep
gviz_ndep = pkgndep::pkgndep(package = "Gviz")
```

```
## retrieve package database from CRAN/Bioconductor (3.17)...
##   - 23632 remote packages on CRAN/Bioconductor.
##   - 324 packages installed locally.
## prepare dependency table...
## prepare reverse dependency table...
```

```
kp_ndep = pkgndep::pkgndep(package = "karyoploteR")
tp_ndep = pkgndep::pkgndep(package = "trackplot")
```

```
par(mar = c(4, 4, 2, 4))

b = barplot(
  height = c(
    gviz_ndep$n_by_strong,
    kp_ndep$n_by_strong,
    tp_ndep$n_by_strong
  ),
  horiz = TRUE,
  names.arg = c("Gviz", "karyoploteR", "trackplot"),
  xlab = "No. of dependencies",
  border = NA,
  col = "#34495e"
)

text(
  c(
    gviz_ndep$n_by_strong,
    kp_ndep$n_by_strong,
    tp_ndep$n_by_strong
  ),
  y = b[, 1],
  labels = c(
    gviz_ndep$n_by_strong,
    kp_ndep$n_by_strong,
    tp_ndep$n_by_strong
  ),
  xpd = TRUE,
  pos = 4
)
```

## Profile plots and heatmaps

### Download datset

Example data from GSE99171
where U87 cell lines are treated with DMSO (control) and dBET6 - a
bromodomain protein degrader. Binding sites for BRD4 transcription
factors are compared at 2 hour and 24 hours post treamnet.

```
dir.create(path = "data/U87/", showWarnings = FALSE, recursive = TRUE)
options(timeout = max(300, getOption("timeout")))

download.file(url = "https://ftp.ncbi.nlm.nih.gov/geo/samples/GSM2634nnn/GSM2634756/suppl/GSM2634756%5FU87%5FBRD4%2Ebw", destfile = "data/U87/BRD4.bw")
download.file(url = "https://ftp.ncbi.nlm.nih.gov/geo/samples/GSM2634nnn/GSM2634756/suppl/GSM2634756%5FU87%5FBRD4%5Fpeaks%2EnarrowPeak%2Egz", destfile = "data/U87/BRD4.bed.gz")
download.file(url = "https://ftp.ncbi.nlm.nih.gov/geo/samples/GSM2634nnn/GSM2634758/suppl/GSM2634758%5FU87%5FBRD4%5FdBET%5F2h%2Ebw", destfile = "data/U87/BRD4_dBET_2h.bw")
download.file(url = "https://ftp.ncbi.nlm.nih.gov/geo/samples/GSM2634nnn/GSM2634757/suppl/GSM2634757%5FU87%5FBRD4%5FdBET%5F24h%2Ebw", destfile = "data/U87/BRD4_dBET_24h.bw")
```

```
u87_bigWigs = list.files(path = "data/U87/", pattern = "\\.bw$", full.names = TRUE)
u87_bigWigs = read_coldata(bws = u87_bigWigs, build = "hg19")
```

```
## Checking for files..
```

```
## Input type: bw
```

```
## Ref genome: hg19
```

```
## OK!
```

```
u87_bigWigs = u87_bigWigs[order(bw_sample_names)] #Order by sample names
u87_bigWigs
```

```
##                      bw_files bw_sample_names
##                        <char>          <char>
## 1:          data/U87//BRD4.bw            BRD4
## 2: data/U87//BRD4_dBET_24h.bw   BRD4_dBET_24h
## 3:  data/U87//BRD4_dBET_2h.bw    BRD4_dBET_2h
```

### Profile plot

```
profile_data = profile_extract(colData = u87_bigWigs, bed = "data/U87/BRD4.bed.gz", startFrom = "center", up = 1500, down = 1500)
```

```
## Extracting signals..
```

```
profile_summarize(sig_list = profile_data, stat = "mean") |> profile_plot()
```

```
## Summarizing..
```

### Heatmap

```
profile_heatmap(mat_list = profile_data, zmaxs = 0.81, top_profile = TRUE)
```

## Benchmark against deeptools

Requires deeptools
and hyperfine to be
installed.

`benchmark/bm_deeptools.sh` and
`benchmark/bm_trackplot.R` contain equivalent code to
generate profile plots and heatmaps from the above U87 data. These two
scripts are benchmarked with hyperfine and results are written to
`benchmark/dt_vs_tp_hf.csv`

```
#Deeptools commands for computeMatrix, plotProfile and plotHeatmap
cat benchmark/bm_deeptools.sh
```

```
## #!/usr/bin/env bash
## 
## bed="data/U87/BRD4.bed"
## outmat="GSM2634761_U87_H3K4Me3_peaks.mat.gz"
## 
## computeMatrix reference-point -R ${bed} --binSize 10 \
## -S data/U87/BRD4.bw \
## data/U87/BRD4_dBET_2h.bw \
## data/U87/BRD4_dBET_24h.bw \
## --outFileName ${outmat} --referencePoint center -a 1500 -b 1500 --samplesLabel DMSO dBET_24h dBET_2h \
## --numberOfProcessors 1
## 
## plotProfile --matrixFile ${outmat} --outFileName ${outmat}.profilePlot.png
## plotHeatmap --matrixFile ${outmat} --outFileName ${outmat}.heatmap.png
```

```
#Trackplot commands
cat benchmark/bm_trackplot.R
```

```
## #!/usr/bin/env Rscript
## 
## source("trackplot.R")
## 
## bws = c("data/U87/BRD4.bw", "data/U87/BRD4_dBET_2h.bw", "data/U87/BRD4_dBET_24h.bw")
## bed = "data/U87/BRD4.bed.gz"
## 
## bws = read_coldata(bws = bws, sample_names = c("DMSO", "dBET_24h", "dBET_2h"), build = "hg19")
## #Center and extend 1500 both ways from the peak center. Estimate mean signal
## pe_bed = profile_extract(colData = bws, bed = bed, startFrom = "center", up = 1500, down = 1500, nthreads = 1)
## pe_bed_sum = profile_summarize(sig_list = pe_bed) 
## 
## #Profile plot
## png("profile_plot.png")
## profile_plot(pe_bed_sum)
## dev.off()
## 
## #Heatmap
## png("heatmap_plot.png")
## profile_heatmap(pe_bed, zmaxs = 0.8)
## dev.off()
```

### Run benchmark

```
hyperfine --export-csv benchmark/dt_vs_tp_hf.csv --warmup 2 -m 3 -M 5 -n deeptools -n trackplot benchmark/bm_deeptools.sh benchmark/bm_trackplot.R
```

```
#Benchmark results
bm = data.table::fread(input = "benchmark/dt_vs_tp_hf.csv")
print(bm)
```

```
##      command      mean      stddev    median      user    system       min
##       <char>     <num>       <num>     <num>     <num>     <num>     <num>
## 1: deeptools 63.996441 0.164012680 63.997244 62.727464 1.0712371 63.832029
## 2: trackplot  2.167876 0.008172938  2.168397  1.887465 0.2184214  2.159455
##          max
##        <num>
## 1: 64.160051
## 2:  2.175776
```

```
par(mar = c(4, 3, 1, 4))
b2 = barplot(bm$mean, horiz = TRUE, names.arg = c("deeptools", "trackplot"), xlab = "Time taken (seconds)", border = NA, col = "#34495e")
text(bm$mean, y = b2[,1], labels = round(bm$mean, 2), xpd = TRUE, pos = 4)
```

## PCA

`trackplot` can be used to extract signal intensities for
genomic regions of interest and perform PC analysis.

***NOTE*** Make sure the bigWig files are
properly normalized and comparable across the samples.

Example data from GSE231425
with H3K27ac signals from hematopoietic stem and, myeloid progenitor
cells.

```
dir.create(path = "data/HSC/", showWarnings = FALSE, recursive = TRUE)
options(timeout = max(300, getOption("timeout")))

download.file(url = "https://ftp.ncbi.nlm.nih.gov/geo/samples/GSM7277nnn/GSM7277506/suppl/GSM7277506_Coverage_H3K27ac_HSC.bw", destfile = "data/HSC/HSC.bw")
download.file(url = "https://ftp.ncbi.nlm.nih.gov/geo/samples/GSM7277nnn/GSM7277507/suppl/GSM7277507_Coverage_H3K27ac_CMP.bw", destfile = "data/HSC/CMP.bw")
download.file(url = "https://ftp.ncbi.nlm.nih.gov/geo/samples/GSM7277nnn/GSM7277508/suppl/GSM7277508_Coverage_H3K27ac_GMP.bw", destfile = "data/HSC/GMP.bw")
download.file(url = "https://ftp.ncbi.nlm.nih.gov/geo/samples/GSM7277nnn/GSM7277509/suppl/GSM7277509_Coverage_H3K27ac_MEP.bw", destfile = "data/HSC/MEP.bw")
```

Prepare input data

```
hsc_bws = list.files(path = "data/HSC/", pattern = "bw", full.names = TRUE)
hsc_cd = read_coldata(bws = hsc_bws, build = "hg38")
```

```
## Checking for files..
```

```
## Input type: bw
```

```
## Ref genome: hg38
```

```
## OK!
```

Extract summary for all protein coding genes (1200bp up and 800 down
of TSS)

```
hsc_summary = extract_summary(colData = hsc_cd, up = 1200, down = 800, pc_genes = TRUE)
```

```
## No bed file was given. Defaulting to ucsc refseq..
```

```
## Extracting gene models from UCSC:
##     build: hg38
##     query: mysql --user genome --host genome-mysql.soe.ucsc.edu -NAD hg38 -e 'select chrom, txStart, txEnd, strand, name, name2 from refGene'
```

```
## Fetched 59701 transcripts from 24 contigs
```

```
## Extracting summaries..
```

Plot PCA

```
pca_plot(summary_list = hsc_summary, size = 1.2, log2 = TRUE, show_cree = FALSE)
```

## SessionInfo

```
sessionInfo()
```

```
## R version 4.3.2 (2023-10-31)
## Platform: x86_64-apple-darwin20 (64-bit)
## Running under: macOS Sonoma 14.2.1
## 
## Matrix products: default
## BLAS:   /Library/Frameworks/R.framework/Versions/4.3-x86_64/Resources/lib/libRblas.0.dylib 
## LAPACK: /Library/Frameworks/R.framework/Versions/4.3-x86_64/Resources/lib/libRlapack.dylib;  LAPACK version 3.11.0
## 
## locale:
## [1] en_US.UTF-8/en_US.UTF-8/en_US.UTF-8/C/en_US.UTF-8/en_US.UTF-8
## 
## time zone: Europe/Berlin
## tzcode source: internal
## 
## attached base packages:
## [1] stats     graphics  grDevices utils     datasets  methods   base     
## 
## other attached packages:
## [1] pkgndep_1.99.3    data.table_1.15.0
## 
## loaded via a namespace (and not attached):
##  [1] ComplexHeatmap_2.18.0 jsonlite_1.8.8        compiler_4.3.2       
##  [4] rjson_0.2.21          highr_0.10            crayon_1.5.2         
##  [7] parallel_4.3.2        cluster_2.1.6         jquerylib_0.1.4      
## [10] IRanges_2.36.0        png_0.1-8             yaml_2.3.8           
## [13] fastmap_1.1.1         R6_2.5.1              shape_1.4.6          
## [16] knitr_1.45            BiocGenerics_0.48.1   iterators_1.0.14     
## [19] GetoptLong_1.0.5      trackplot_1.5.10      circlize_0.4.15      
## [22] RColorBrewer_1.1-3    bslib_0.6.1           R.utils_2.12.3       
## [25] rlang_1.1.3           cachem_1.0.8          xfun_0.41            
## [28] sass_0.4.8            GlobalOptions_0.1.2   doParallel_1.0.17    
## [31] cli_3.6.2             digest_0.6.34         foreach_1.5.2        
## [34] grid_4.3.2            rstudioapi_0.15.0     clue_0.3-65          
## [37] lifecycle_1.0.4       R.methodsS3_1.8.2     R.oo_1.26.0          
## [40] S4Vectors_0.40.2      evaluate_0.23         codetools_0.2-19     
## [43] hash_2.2.6.3          stats4_4.3.2          colorspace_2.1-0     
## [46] BiocVersion_3.18.1    rmarkdown_2.25        matrixStats_1.2.0    
## [49] tools_4.3.2           htmltools_0.5.7
```
